# Influence of Individual Differences in fMRI-Based Pain Prediction Models on Between-Individual Prediction Performance

**DOI:** 10.3389/fnins.2018.00569

**Published:** 2018-08-15

**Authors:** Qianqian Lin, Linling Li, Jia Liu, Weixiang Liu, Gan Huang, Zhiguo Zhang

**Affiliations:** ^1^School of Biomedical Engineering, Health Science Center, Shenzhen University, Shenzhen, China; ^2^Guangdong Provincial Key Laboratory of Biomedical Measurements and Ultrasound Imaging, Shenzhen University, Shenzhen, China

**Keywords:** pain prediction, individual difference, fMRI decoding, machine learning, between-individual prediction

## Abstract

Decoding subjective pain perception from functional magnetic resonance imaging (fMRI) data using machine learning technique is gaining a growing interest. Despite the well-documented individual differences in pain experience and brain responses, it still remains unclear how and to what extent these individual differences affect the performance of between-individual fMRI-based pain prediction. The present study is aimed to examine the relationship between individual differences in pain prediction models and between-individual prediction error, and, further, to identify brain regions that contribute to between-individual prediction error. To this end, we collected and analyzed fMRI data and pain ratings in a laser-evoked pain experiment. By correlating different types of individual difference metrics with between-individual prediction error, we are able to quantify the influence of these individual differences on prediction performance and reveal a set of brain regions whose activities are related to prediction error. Interestingly, we found that the precuneus, which does not have predictive capability to pain, could also affect the prediction error. This study elucidates the influence of interindividual variability in pain on the between-individual prediction performance, and the results will be useful for the design of more accurate and robust fMRI-based pain prediction models.

## Introduction

Pain is a subjective unpleasant experience (Loeser and Treede, 2008). Self-report is the golden standard in clinical applications. Studies have found that physiological signatures of pain could be used to develop new pain assessment tools that complement self-report (Wager et al., 2013; Woo et al., 2017; Reddan and Wager, 2018). Thus, identifying objective physiological signatures of pain is highly desired in clinical practice and basic research. Functional magnetic resonance imaging (fMRI) studies have showed that painful nociceptive stimuli evoked blood oxygen level dependent (BOLD) responses in the “pain matrix,” which includes the primary somatosensory cortex, anterior cingulate cortex, and insula, etc. (Ingvar, 1999; Iannetti and Mouraux, 2010; Legrain et al., 2011). Consequently, decoding an individual’s subjective pain perception from BOLD responses in the “pain matrix” is considered to be a potential and promising pain assessment technique (Marquand et al., 2010; Brown et al., 2011; Brodersen et al., 2012; Schulz et al., 2012).

Basically, fMRI-based pain decoding has two phases: (1) training a prediction model from a group of individuals with labeled data (single-trial pain-evoked BOLD responses with corresponding pain ratings), (2) applying the pain prediction model to pain-evoked BOLD responses from another group of individuals. Such a prediction scheme is referred to as “between-individual prediction,” which is in contrast to “within-individual prediction” (i.e., to train and apply a model in the same group of individuals) (Tu et al., 2016). Studies have shown that remarkable individual differences in subjective pain perception as well as in neural responses could lower the accuracy of between-individual pain prediction (Huang et al., 2013; Hu and Iannetti, 2016). Various advanced machine learning techniques, such as multi-task learning (Marquand et al., 2014) and random-forest (Vijayakumar et al., 2017), have been used to boost the performance of between-individual pain prediction. In Lindquist et al. (2017), group-level priors were incorporated into individual prediction models to improve their accuracy when being used for new individuals. In an electroencephalographical (EEG) study (Bai et al., 2016), one individual’s pain-evoked EEG responses were normalized by this individual’s rest EEG to reduce the individual difference in EEG features, which can significantly increase the accuracy of between-individual prediction.

All above new prediction methods attempted to build a more robust and general between-individual prediction model by minimizing the differences in neural features or prediction models. These methods do have improved the accuracy of between-individual pain prediction to some extent, but their performance and applications are still limited. A key reason underlying this limitation is the little knowledge about the relationship between individual differences in fMRI-based prediction models and between-individual prediction performance. Actually, an in-depth examination on how and to what extent these individual differences synergistically determine the between-individual prediction accuracy is still lacking. For instance, it still remains unclear whether only those pain-related regions (such as the pain matrix) determine the between-individual prediction error, or other cortical regions also play a role. A thorough investigation of the relationship between various types of individual differences and between-individual prediction error is of key importance. It can provide meaningful information about the neural mechanisms of pain perception at the individual level, and can be useful in guiding the design of a better between-individual pain prediction model.

The present study is aimed to quantitatively analyze the relationship between individual differences in pain prediction models and between-individual prediction error, and more specifically, to identify cortical regions that determine the prediction error of between-individual prediction. To achieve this, we collected fMRI data and pain ratings in a laser-evoked pain experiment and measured the individual differences in subjective pain ratings and in BOLD responses. Then we trained a prediction model from single-trial BOLD responses and ratings for each individual and applied this model to another individual. Prediction error metrics were calculated and correlated with different types of individual difference metrics to quantify how and to what extent these individual difference metrics determine between-individual prediction performance.

## Materials and Methods

### Experiments

This study included 32 healthy participants (20 females), aged years 22.1 ± 2.0 (mean ± SD). The initial inclusion and exclusion criteria were based on the general health questionnaire, pain safety screening form, and fMRI safety screening form. Participants reported no history of chronic pain, psychiatric, or neurological disorders. The experimental procedures were approved by the local ethics committee (Approval No. SWU20140607). All participants gave written informed consent. They were familiarized with the experiment paradigm before the experiment. Painful laser stimuli were delivered to participants when they were lying in the MRI scanner and fMRI scans were on. Radiant-heat stimulus energies included four levels (E1: 2.5J; E2: 3.0J; E3: 3.5J; E4: 4.0J), and 10 laser pulses at each of the four energies were delivered in a random order on the dorsum of the left hand, with a total of 40 pulses per individual. The duration of a laser stimulus was 0.5 s. The inter-stimulus interval ranged from 27 to 33 s (uniformly distributed), which consisted of two periods: the time interval between one stimulus and its rating was 15–18 s (uniformly distributed) and the time interval between the rating and the next stimulus was 9–18 s (uniformly distributed). Participants reported pain intensity using a visual analog scale ranging from 0 to 10 (0: no pain; 10: pain as bad as it could be). The total duration of the experiment was around 25 min. Two participants were excluded from subsequent analyses because they did not show large variations in pain perception. More precisely, these two participants had a small range of pain ratings (<4) and were regarded as outliers, while other 30 participants had a range of pain ratings 8.2 ± 1.5 (mean ± SD). Different analyses of the same dataset were published in Tu et al. (2016, 2018).

Magnetic resonance imaging (MRI) data were collected using a Siemens 3.0 Tesla Trio scanner with a standard head coil. Functional images were acquired with echo planar imaging (EPI) sequence with the following parameters: 255 mm thick slices and 0.5 mm inter-slice gaps, TR = 1500 ms, TE = 29 ms, filed of view = 192 mm × 192 mm, 64 × 64 matrix, 3 mm × 3 mm × 3 mm voxels, flip angle = 90°. A high-resolution T1-weighted structural image (1 mm^3^ isotropic voxel MPRAGE) was acquired after functional imaging.

### fMRI Data Preprocessing and Feature Extraction

Functional MR images were analyzed using SPM8^1^. For each participant, fMRI data were slice-timing corrected, head motion corrected, normalized to the Montreal Neurological Institute (MNI) space (voxel size = 3 × 3 × 3) by mapping T1-weighted structural images to the MNI template, and then smoothed with an 8 mm FWHM Gaussian kernel. A high-pass filter was applied (cut-off frequency = 1/128 Hz) to the BOLD time-series to remove low-frequency drifts. BOLD responses were normalized by subtracting and then dividing their baseline values at stimulus onset (Brown et al., 2011). The maximum BOLD responses (4^th^ scan after stimulus onset) were extracted as fMRI features for prediction of subjective pain ratings.

### Identification of Pain Predictive Brain Regions

We used partial least squares regression (PLSR) (Hu et al., 2014) to model the trial-by-trial relationship between whole-brain BOLD features and pain ratings for each individual. PLSR can well address the problems of high dimensionality and multicollinearity of fMRI features and is a suitable model for pain prediction (Tu et al., 2016). For the *j*-th individual, the PLSR model is formulated as:

(1) $$R_{\text{i}}^{\left(j\right)}=B_{\text{i}}^{\left(j\right)}a^{\left(j\right)}$$

where R_i_^(j)^ is the pain rating of the *i*-th trial, B_i_^(j)^ is the whole-brain BOLD features (the maximal responses at the 4^th^ scan after stimulus onset) of the *i*-th trial, and a^(j)^ is the PLSR model coefficient vector. Each coefficient in the PLSR coefficient vector a^(j)^ represents the predictive capability of the pain-evoked BOLD responses at the corresponding voxel. The SIMPLS algorithm was used to compute the PLSR model coefficients (De Jong, 1993). SIMPLS first constructed a set of linear combinations of the inputs, which are known as the latent variables, in order to reduce the dimensionality of data. Then, the response variables would be regressed on these latent variables. The number of latent components in the PLSR analysis was estimated using the coefficient of determination, which calculates the percentage of the variance of the values fitted by the latent components and the total variance of the dependent variables. A Matlab function “plsregress”^2^ was used for the implementation of PLSR in MatlabR2018a (MathWorks, Natick, MA, United States).

In order to identify pain predictive brain regions at the group level, we further used a one-sample *t*-test against zero to assess the significance of these model coefficients across individuals. To account for multiple comparisons, the significance level was corrected using family-wise error (FWE) rate (Hochberg, 1988). Hereinafter, brain regions whose coefficients were significantly different from zero were referred to as “predictive regions,” while other regions were “non-predictive regions.”

### Quantification of Between-Individual Prediction Error

In the scheme of between-individual pain prediction, BOLD responses and pain ratings of all trials from one individual were used to train a prediction model using PLSR, and this model was then applied on BOLD responses of all trials from another participant. Because we had in total 30 participants, the individual-by-individual pain prediction scheme resulted in 900 pairwise individual-by-individual pain prediction results, 870 of which were between-individual prediction and 30 of which were within-individual prediction. The prediction error of each pair of individuals was measured by two metrics: mean absolute error (MAE) and mean prediction bias (MPB). MAE and MPB are respectively calculated as:

(2) $$MAE_{\text{t,s}}=\frac{1}{I}\sum_{\text{i}=1}^{\text{I}}\left|{\hat{R}}_{\text{i}}^{\left(\text{t;s}\right)}-R_{i}^{\left(t\right)}\right|$$

(3) $$MPB_{\text{t,s}}=\frac{1}{I}\sum_{\text{i}=1}^{\text{I}}\left({\hat{R}}_{\text{i}}^{\left(\text{t;s}\right)}-R_{i}^{\left(t\right)}\right)$$

where R_i_^(t)^ is the true pain rating of the *i*-th trial of the *t*-th individual, ${\hat{R}}_{\text{i}}^{\left(\text{t;s}\right)}$ is the corresponding predicted pain rating estimated from the model trained from the *s*-th individual, and *I* is the total number of trials of the *t*-th individual. Note that, MAE and MPB are related but represent different aspects of the prediction error. MAE is a positive number and is a measure of the overall distance between predicated and true values. On the other hand, MPB is a signed number: a positive MPB means the predicted pain rating is greater than the actual value and a negative MPB means the predicted value is smaller than the actual value. In another word, MPB denotes an overall increase or decrease of predicated pain ratings, with respect to actual pain ratings. We also compared these two prediction error metrics between between-individual prediction (870 pairs) and within-individual prediction (30 pairs) using a two-sample *t*-test.

### Quantification of Individual Differences in Pain Prediction Models

There were two types of individual differences in a pain prediction model and they were respectively calculated from class labels (i.e., pain ratings, R in the prediction model) and features (i.e., pain-evoke BOLD responses, B in the prediction model). For each type of individual differences, we can calculate two individual difference metrics: the distance (a positive number) between the training individual and the test individual and the difference (a signed number) by subtracting values of the training individual from values of the test individual. The Bhattacharyya distance was used to calculate the distance between the training individual and the test individual. Bhattacharyya distance can reflect the degree of dissimilarity between any two probability distributions and is more reliable than, for example, Mahalanobis distance (Bhattacharyya, 1943; Ali and Silvey, 1966). Bhattacharyya distance has been very popularly used in a wide range of biomedical and engineering applications, such as feature extraction of medical signals (Taghizadeh-Sarabi et al., 2015), image segmentation (Ning et al., 2010) and speech recognition (Lee et al., 2011). For each pair of individuals, we calculated the following four individual difference metrics.

1. Distance of pain ratings (DIST_PAIN_): calculated as the Bhattacharyya distance of pain ratings between multiple trials of two individuals;
2. Difference of pain ratings (DIFF_PAIN_): calculated as the difference of across-trial averaged pain ratings between two individuals (test individual – training individual);
3. Distance of BOLD responses (DIST_BOLD_): at each voxel, calculated as the Bhattacharyya distance of normalized BOLD features between multiple trials of two individuals;
4. Difference of BOLD responses (DIFF_BOLD_): at each voxel, calculated as the difference of across-trial averaged normalized BOLD features between two individuals (test individual – training individual).

### Relationship Between Individual Differences and Prediction Error

Subsequently, we explored the relationship between the individual difference metrics (DIST_PAIN_, DIFF_PAIN_, DIST_BOLD_, DIFF_BOLD_) and prediction error metrics (MAE and MPE), with the aim to identify how individual differences in pain prediction models determine the between-individual prediction error. To this end, we correlated two sets of unsigned metrics (i.e., DIST_PAIN_/DIST_BOLD_ and MAE) and correlated two sets of signed metrics (i.e., DIFF_PAIN_/DIFF_BOLD_ and MPB) by calculating the Pearson’s correlation coefficients across 870 pairs of different individuals. It should be noted that, the correlation analyses between prediction error metrics and individual difference metrics of BOLD responses were carried out in a voxel-wise manner. The multiple comparisons problem was corrected by using FWE.

## Results

### Behavioral Data Analyses

**FIGURE 1:**
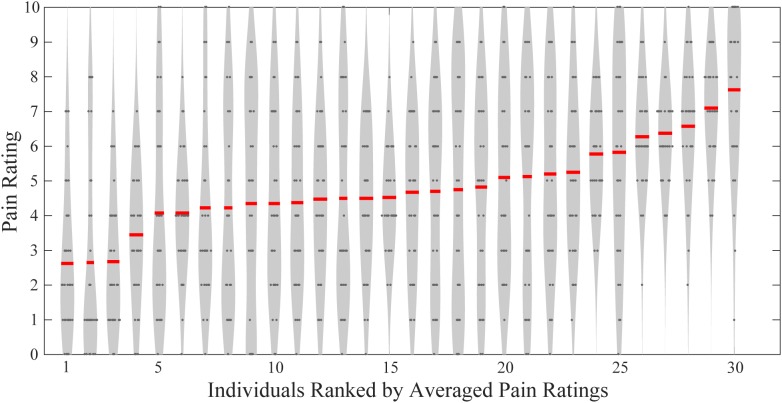
Individual pain ratings of all participants. Gray dots indicate single-trial pain ratings, and red lines represent individuals’ averaged pain ratings.

As shown in **Figure 1**, these participants had largely different levels of pain perception in responses to the same set of painful stimuli. Averaged pain ratings ranged from 2.63 ± 2.11 to 7.63 ± 2.23. Using Pearson’s correlation analyses, we found that neither the age nor the weight was significantly correlated with the averaged pain ratings (age and rating: *R* = -0.175, *P* = 0.356; weight and rating: *R* = 0.0978, *P* = 0.607). Also, males and females did not have significant difference in their averaged pain ratings (two-sample *t*-test, *P* = 0.428). Therefore, age, weight, and gender cannot explain between-individual differences in subjective pain ratings in the present study. But the number of participants in this study is still small and a large-sample study should be carried to produce more significantly powerful results.

### Between-Individual Pain Prediction Error

**FIGURE 2:**
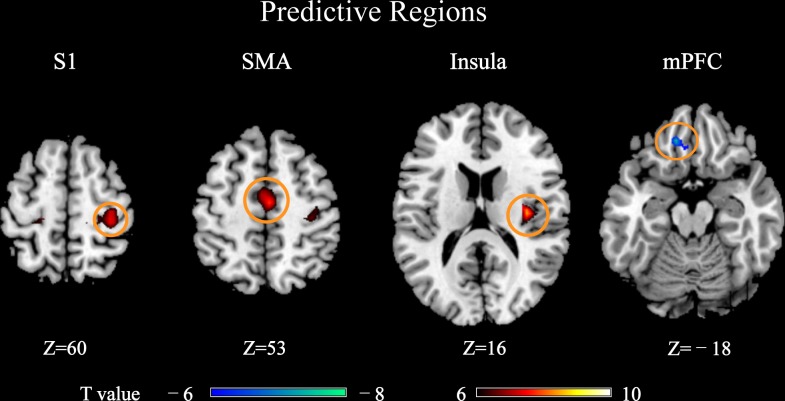
Brain regions whose pain-evoked BOLD responses (4th scan after stimulus onset) were predictive of subjective pain ratings in PLSR models (P_FWE_ < 0.05). These regions were called “predictive regions,” while other regions were “non-predictive regions.” Brain regions: S1, primary somatosensory cortex; SMA, supplementary motor area; mPFC, medial prefrontal cortex.

The PLSR analyses revealed that BOLD responses within a wide range of brain regions were predictive of pain perception, as shown in **Figure 2**. These predictive regions included the primary somatosensory cortex (S1), supplementary motor area (SMA), medial prefrontal cortex (mPFC) and insula, all of which were well-documented in literature as the pain-related regions. Among these predictive regions, S1, SMA, and insula had BOLD responses that were positively predictive of pain ratings and only the mPFC had BOLD responses that were negatively predictive of pain ratings. Hence, S1, SMA, and insula were also called as positively predictive regions, while mPFC was a negatively predictive region.

**FIGURE 3:**
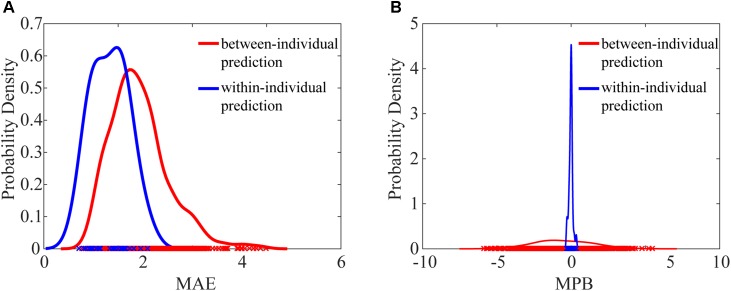
Comparison of probability density functions of MAE **(A)** and MPB **(B)** between within-individual prediction and between-individual prediction. MAE values of between-individual prediction and within-individual prediction were significantly different (*P* = 5.06 × 10^-8^, paired *t*-test), and MPB values of between-individual prediction and within-individual prediction were also significantly different (*P* = 0.038, paired *t*-test).

We next compared the prediction error metrics, MAE and MPB, between within-individual prediction and between-individual prediction. The probability density functions of MAE and MPB were approximated using kernel density estimation (Parzen, 1962). As shown in **Figure 3**, both MAE and MPB were significantly different between two prediction schemes. Between-individual prediction had greater MAE values and greater MPB values (in the magnitude) than within-individual prediction (MAE: *P* = 5.06 × 10^-8^; MPB: *P* = 0.038). Within-individual prediction had very small MPB close to zero (-0.023 ± 0.13), while MPB of between-individual prediction was much dispersed (-0.53 ± 1.29).

**FIGURE 4:**
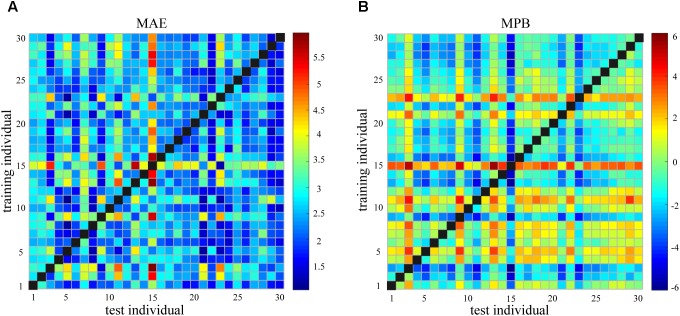
**(A)** Mean absolute error (MAE) of between-individual pain prediction for all pairs of individuals. **(B)** Mean prediction bias (MPB) of between-individual pain prediction for all pairs of individuals.

**Figure 4** shows the pairwise between-individual prediction error as measured by MAE and MPB. It can be seen that the between-individual prediction error was generally large and widely dispersed (MAE: 2.57 ± 0.80; MPB: -0.53 ± 1.29). If MPB is either too large or too small (or say, MPB is large in the magnitude), the corresponding MAE will be large.

### Relationship Between Individual Difference in Pain Ratings and Prediction Error

**FIGURE 5:**
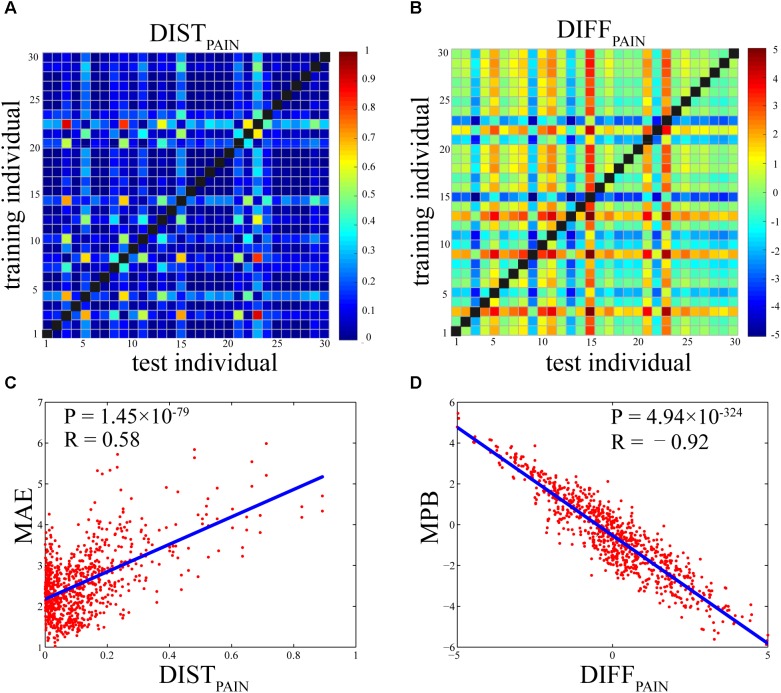
**(A)** Individual differences in pain ratings as measured by DIST_PAIN_ (the Bhattacharyya distance of pain ratings between two individuals). **(B)** Individual differences in pain ratings as measured by DIFF_PAIN_ [the difference (test-training) of across-trial averaged pain ratings between two individuals]. **(C)** The Pearson’s correlation coefficient between DIST_PAIN_ and MAE (*R* = 0.58, *P* = 1.45 × 10^-79^). **(D)** The Pearson’s correlation coefficient between DIFF_PAIN_ and MPB (*R* = –0.92, *P* = 4.94 × 10^-324^). Each dot in **(C)** or **(D)** represents one pair of individuals and there are totally 870 pairs.

**Figures 5A,B** show the DIST_PAIN_ matrix and the DIFF_PAIN_ matrix, respectively. It can be seen that most values in these two matrices were in a relatively small range (DIST_PAIN_: 0.12 ± 0.14; DIFF_PAIN_: 2.04 ± 1.70), but some individuals had extremely large or small pain ratings so that their differences in pain ratings with others were pronounced (shown as some rows and columns with largely different colors). **Figures 5C,D** show that, the relationship between DIST_PAIN_ and MAE and the relationship between DIFF_PAIN_ and MPB were both very significant (DIST_PAIN_ and MAE: *R* = 0.58, *P* = 1.45 × 10^-79^; DIFF_PAIN_ and MPB: *R* = -0.92, *P* = 4.94 × 10^-324^).

### Relationship Between Individual Differences in BOLD Responses and Prediction Error

**FIGURE 6:**
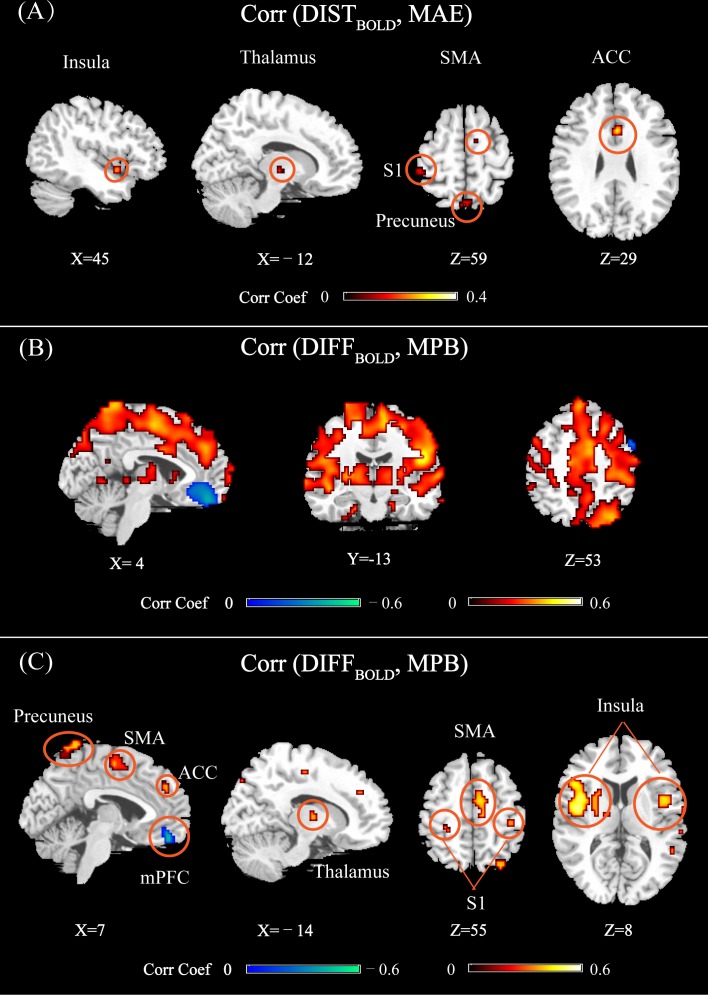
**(A)** Brain regions whose DIST_BOLD_ (calculated as the Bhattacharyya distance of normalized BOLD features between multiple trials of two individuals) were significantly correlated with MAE (P_FWE_ < 0.05). **(B)** Brain regions whose DIFF_BOLD_ [calculated as the difference of across-trial averaged normalized BOLD features between two individuals (test-training)] were significantly correlated with MPB (P_FWE_ < 0.05). **(C)** Brain regions whose DIFF_BOLD_ were significantly correlated with MPB (P_FWE_ < 10^-20^). Brain regions: ACC, anterior cingulate cortex; S1, primary somatosensory cortex; SMA, supplementary motor area; mPFC, medial prefrontal cortex.

**Figure 6** shows the brain regions at which DIST_BOLD_ or DIFF_BOLD_ was significantly correlated with MAE or MPB (using different statistical thresholds for detecting significant regions). In **Figure 6A**, DIST_BOLD_ at thalamus, the anterior cingulate cortex (ACC), S1, SMA, insula, and precuneus were correlated with MAE. In **Figure 6B** (P_FWE_< 0.05) and **Figure 6C** (P_FWE_< 10^-20^), DIFF_BOLD_ at ACC, S1, SMA, insula, thalamus, mPFC, and precuneus were correlated with MPB. Basically, most of the MPB-related regions shown in **Figure 6B** can also be observed in **Figure 6C**. Some regions, such as temporal regions, were also found to be correlated with MPB in **Figure 6B** where P_FWE_< 0.05 was used, though they cannot pass a stricter threshold of P_FWE_< 10^-20^ (as shown in **Figure 6C**).

From **Figure 6**, it can be seen that most of the identified regions were pain predictive regions, except for the precuneus, thalamus, and ACC. ACC and thalamus were not identified as pain-predictive in **Figure 2**, but they were reported to be pain-predictive in literature (Tu et al., 2016). Actually, these two regions could be identified if we use a larger statistical threshold (for example, by using false distortion rate for multiple comparisons correction; not shown here), but they did not pass a stricter FWE-corrected threshold (P_FWE_= 0.05), which may be due to the limited number of participants in the experiment. We can also see that, the regions identified in **Figures 6A,C** were largely overlapped, except for that mPFC was not identified in **Figure 6A**. The absence of mPFC in **Figure 6A** may be due to limited data samples and less statistical power. Actually, by setting a larger threshold (for example, by using false distortion rate), mPFC can also be observed in **Figure 6A**. Overall, by comparing **Figures 6A,B**, we can see that there were more and larger brain regions whose DIFF_BOLD_ was correlated with MPB than those regions whose DIST_BOLD_ was correlated with MAE.

Furthermore, we can see from **Figure 6A** that all identified regions were positively correlated with MAE. However, the brain regions shown in **Figure 6C** could be either positively or negatively correlated with MPB. At positive predictive regions (such as S1, SMA, insula, thalamus, and ACC), DIFF_BOLD_ was positively correlated with MPB. On the contrary, at the negatively predictive region (i.e., mPFC), DIFF_BOLD_ was negatively correlated with MPB.

Importantly, both **Figures 6A,C** revealed that the precuneus was related to between-individual prediction error. If the individual variability in BOLD responses as measured by DIST_BOLD_ or DIFF_BOLD_ at the precuneus is larger, both MAE and MPB will be larger. However, it is interesting to note that, the precuneus was actually not predictive of subjective pain perception (averaged PLSR coefficients in this regions are 0.0013 ± 0.014, which are not significantly different from zero). This implies that the precuneus may modulate pain perception in an indirect manner, which will be discussed in the next section.

## Discussion

It is well-known that the accuracy of between-individual prediction is usually lower because of significant individual differences in the subjective pain perception and neural responses, but it remains unclear how and to what extent these individual differences determine the between-individual prediction error. In the present study, we performed an in-depth examination of the relationship between individual variations in fMRI-based pain prediction models and between-individual prediction error. We found that the between-individual prediction error was mainly determined by individual variability in pain rating and was also affected by individual differences in BOLD responses at a set of specific brain regions.

### Between-Individual Pain Prediction in the Context of Machine Learning

In the context of machine learning, pain ratings and BOLD responses are respectively the class labels and features in the prediction model linking whole-brain BOLD features to pain ratings on a single-trial basis. Pain ratings and BOLD responses of the training individual constitute a source domain, while pain ratings and BOLD responses of the test individual constitute a target domain. Whether a model trained in the source domain can achieve good performance in the target domain is the central issue studied in transfer learning (Pan and Yang, 2010), which is an important branch of machine learning. If the class labels or features in the source domain and target domain have different distributions, applying a model trained in the source domain to the target domain will normally end up with low performance.

Basically, different individuals have different pain prediction models because their pain feelings and brain activities have different distributions, which could be caused by a wide range of factors, such as sociocultural variables and genetic constitution (Nielsen et al., 2009). Either individual differences in class labels or individual differences in features will lead to different prediction models for individuals, and then cause between-individual prediction error. Based on the PLSR models used, the results on the correlations between individual difference metrics and prediction error metrics could be interpreted as follows.

First, we consider the influence of individual variability in pain ratings on prediction error. Suppose the test and trainings individuals have the same distributions for BOLD responses, and, without loss of generality, assume the test individual has higher pain ratings than the training individual, which means DIFF_PAIN_ is positive. Thus, the overall predictive capability of the whole-brain BOLD responses of the test individual should be larger than that of the training individual, which makes the test individual have higher pain ratings. If we apply the model trained in the training individual to the test individual, the predicted pain ratings should be lower than the actual ones, resulting in a negative MPB. As a result, the correlation between DIFF_PAIN_ and MPB is negative, which can be clearly seen in **Figure 5D**.

Second, we discuss the influence of individual variations in BOLD responses on prediction error. Suppose the test and training individuals have the same distributions for pain ratings, and, without loss of generality, assume the test individual has stronger BOLD responses within the positively predictive regions (SMA, S1, insula) than the training individual, which means DIFF_BOLD_ is positive. Because the training and test individuals have the same pain ratings, the predictive capability of BOLD within the positively predictive regions of the training individual should be larger than that of the test individual. If we apply the model trained in the training individual to the test individual, the predicted pain should be greater than the actual ones, resulting in a positive MPB. As a result, the correlation between DIFF_PAIN_ and MPB is positive. Similar interpretation holds true for negatively predictive regions (i.e., mPFC).

### Patterns of Influences of Individual Differences in Pain Prediction Models on Prediction Error

We now elucidate how individual differences in pain prediction models influence between-individual prediction error. In short, individual differences in pain ratings and individual differences in BOLD responses jointly determined the between-individual prediction error: larger the individual differences, larger the prediction error.

From the results that DIST_PAIN_ is positively correlated with MAE and DIFF_PAIN_ is negatively correlated with MPB, we can see that large between-individual difference in pain ratings leads to large prediction error. More precisely, if the test individual has higher pain ratings than the training individual, then the predicted pain ratings are lower than the actual ratings.

The patterns of influences of individual differences in BOLD responses on between-individual prediction error are more complicated and exhibit spatial heterogeneity. At thalamus, ACC, S1, SMA, insula, and precuneus, large between-individual distance in BOLD responses leads to large prediction error, because at these regions DIST_BOLD_ are positively correlated with MAE and DIFF_BOLD_ are positively correlated with MPB. Specifically, if the BOLD responses within these regions of a test individual are larger than those of a training individual, then the predicted pain ratings will be greater than the actual values. On the other hand, if the BOLD responses at the mPFC, where DIFF_BOLD_ is negatively correlated with MPB, of a test individual are larger than those of a training individual, the predicted pain ratings will be lower than the actual values.

Among these individual difference metrics, individual differences in pain ratings are the most important determinants of the between-individual prediction error. It can be seen from the results that the influence of individual differences in BOLD responses on prediction error is much smaller (the correlation coefficients between DIST_BOLD_ and MAE are in the range from 0.1735 to 0.3125 and the correlation coefficients between DIFF_BOLD_ and MPB are in the range from -0.4623 to 0.5500) than the influence of the individual differences in pain ratings on prediction error (the correlation coefficient between DIST_PAIN_ and MAE is 0.5806 and the correlation coefficient between DIFF_PAIN_ and MPB is 0.9186). Because people have largely different pain sensitivity, their feelings of pain can differ significantly, which seriously degrades the performance of between-individual pain prediction. It can also be seen from **Figure 3** that, for within-individual prediction where difference in pain ratings is zero, the estimation bias MPB is close to zero. On the other hand, although variations in BOLD responses are also evident, their influence on prediction error is relatively small.

### Non-predictive Regions Influence Between-Individual Prediction Error

It is not surprising that the individual differences of BOLD responses in those pain predictive regions (S1, SMA, and insula) determine between-individual prediction error, because variances of BOLD responses introduce differences in the feature space for different individuals, as discussed in Section “Between-Individual Pain Prediction in the Context of Machine Learning.” But we also found that a non-predictive region, the precuneus, also affected the prediction error. More precisely, DIFF_BOLD_ and DIST_BOLD_ in the precuneus respectively determined the prediction error in terms of MAE and MBP.

We first discuss the possible functional role of the precuneus in pain perception. Conventionally, the precuneus is not considered as a core region of pain perception, because it is not activated by pain stimulation in various pain experiments (Tu et al., 2016). Although some fMRI studies have found that the precuneus [as part of the default mode network (DMN)] was deactivated by painful stimulation (Atlas et al., 2014) and its activity strength was negatively correlated with pain intensity (Porro et al., 1998), the deactivation results were not consistent in literature (Koyama et al., 2005; Kong et al., 2010). In the present study, we found that the mPFC, which is also an important part of DMN, had negative predictive capability to pain but the precuneus had no predictive capability. However, it is still well-documented that the precuneus is involved in the modulation of pain perception. For instance, an EEG study showed the EEG activation at the precuneus was correlated with the pain sensitivity (Goffaux et al., 2014). It was suggested that, because the precuneus is engaged in continuous information gathering, its activity could be related to the saliency of external stimulation (Goffaux et al., 2014). Saliency of stimulation can largely determine subjective pain perception and evoke brain activities (Iannetti et al., 2008). Therefore, the precuneus activity may modulate subjective pain ratings and neural responses through processing salient events in an individual-specific manner. That is, different individuals have different patterns of saliency detection and evaluation, which leads to different levels of pain experience in response to the same sets of stimulation. As a consequence, individual differences in the precuneus activity could (partially) determine between-individual pain prediction error, even though the precuneus activity is not directly predictive of pain ratings. It is also important to mention that, the modulation effect of the precuneus activity on pain prediction error should not be specific to pain, because it is more closely related to salience processing.

Further, we would like to argue that, the precuneus’s pain-predictive capability does exist for individuals, but it cannot be identified at the group level because this region may modulate pain perception in different directions (i.e., PLSR model coefficients in this region have different signs across individuals). In another word, unlike those pain-predictive regions (S1, SMA, Insula) at which the activities are positively correlated with pain intensity and the positive correlation is consistent across individuals, the precuneus’s BOLD responses could be positively or negatively related to pain perception so its group-level effect is not significant.

Actually, the mean value of the model coefficients in the precuneus (as defined in **Figure 2**) is -0.0011 ± 0.0137, which is not significantly different from zero (*P* = 0.65, one-sample *t*-test) and is much smaller than those of predictive regions as defined in **Figure 2** (S1: 0.043 ± 0.032, *P* = 3.66 × 10^-8^; SMA: 0.035 ± 0.023, *P* = 3.38 × 10^-9^; Insula: 0.026 ± 0.015, *P* = 3.15 × 10^-10^; mPFC: -0.053 ± 0.038, *P* = 2.70 × 10^-8^). But the mean absolute values of model coefficients in the precuneus is 0.0284 ± 0.0072, which is comparable to those of predictive regions (S1: 0.025 ± 0.0095; SMA: 0.026 ± 0.012; Insula: 0.023 ± 0.0082; mPFC: 0.056 ± 0.024). Anyway, it is still not clear why individuals have different directions for the precuneus’s predictive capability.

In addition, it is also possible that the precuneus modulates subjective pain perception as an important part of the DMN, which consists of PCC, mPFC, angular gyrus, etc. Actually, it has been reported that the anatomical and functional features of DMN are correlated with pain sensitivity and pain perception. A structural MRI study reported that individuals with high pain sensitivity have less gray matter in the precuneus and PCC (Emerson et al., 2014). Regarding functional neural correlates of individual differences in pain, fMRI studies have shown that, individual variability in pain could be caused by differences in the levels of attention and expectation to painful stimuli, which are reflected in DMN’s activation or deactivation (Kucyi and Davis, 2015). For example, the DMN exhibited decreased activation in proportion to the intensity of expected pain (Koyama et al., 2005). When attention was focused away from pain, the DMN was engaged and showed pain-induced deactivation (Wiech, 2016). However, we did not find other parts of DMN, such as PCC and angular gyrus, modulate an individual’s pain perception and the between-individual prediction error the same way as the precuneus. Hence, different parts of DMN may have different effects on subjective pain perception, so the role and mechanism of the precuneus in pain perception may not be generalized to other DMN regions.

### Implications for Improving Performance of Between-Individual Pain Prediction

This study revealed how individual differences in subjective pain ratings and in BOLD responses determine the between-individual prediction error. Based on these findings, it is possible to develop new between-individual prediction methods that can overcome the adverse influence of individual differences. One straightforward approach is to minimize the difference of the features or class labels between training and test individuals. Minimizing the difference of feature spaces between test individuals and training individuals is relatively simple and it can be realized by using feature normalization (Bai et al., 2016) or domain adaptation methods (such as covariate shift) (Pan and Yang, 2010). But, it is difficult, or even impossible, to minimize the difference in pain ratings across individuals, because the ratings of test individuals are generally unknown in clinical practice. As discussed earlier, individual differences in pain ratings have a much larger impact on the between-individual prediction error than individual differences in BOLD responses. This implies that, only minimizing the distance of feature spaces may not be able to achieve significantly improved prediction accuracy, because it is the differences in pain ratings that predominantly influence the between-individual predication error.

Another possible approach is to select a subset of training individuals, who have similar pain ratings (or, in general, pain sensitivity) as those of a given test individual, to build a model for this test individual. For example, if a signature of pain sensitivity can be easily measured and we know the test individual has a high sensitivity to pain from this signature, we can then only select individuals with similar levels of pain sensitivity as the test individual to train a prediction model. Optimizing training set could potentially improve the accuracy of the between-subject pain prediction, and it could also reduce the time for data collection and computation.

### Limitations and Future Work

Individual differences in pain experience and brain activities are attributed to a wide range of factors, such as psychophysiological states, genetic variables, experiences, and resting-state brain activity. For example, people in difference mental states (such as drowsy, anxiety, depression) have largely different pain ratings and brain activities. Also, if an individual has ever participated in a similar experiment, his/her performance in the second experiment may be different from the first experiment, because pain is modulated by experience. But this experiment did not record these types of variables and only examined how the individual differences in pain-evoked BOLD signals and pain ratings affect the prediction error. Thus, the determinants of between-individual pain prediction error are still limited in these two types of variables (pain ratings and evoked BOLD signals). It is necessary and possible to collect more types of behavioral and demographic data as well as multi-modal neurophysiological signals to provide a more comprehensive picture of the determinants of between-individual prediction error.

Similarly, subjective pain ratings are not only correlated by pain-evoked BOLD responses, but also related to many other variables (such as gender and sociocultural variables) and neural activities (such as resting-state brain connectivity and pre-stimulus neural oscillations). Including more independent variables (features) in the pain prediction model may improve the prediction accuracy, but it becomes more difficult to disentangle the contributions of various types of features to prediction error because there is complex dependency among these features. In the present study, we built the pain prediction model only using pain-evoked BOLD responses as features to ensure the relationship between individual differences in BOLD features and pain prediction error can be well-elucidated.

Lastly, the number of individuals and the number of trials per individual in this experiment are not large, which limits the statistical power of the results. Here, we have analyzed data from 30 individuals. Increasing the number of individuals will make the data contain more possible variations across individuals, which will make our conclusion more robust and generalized. For example, this study showed that age, weight, and gender were not related to subjective pain ratings. But the number of participants in this study is still small and a large-sample study should be carried out to produce more significantly powerful results. The number of trials per individual is also very important in training a model, because more training trials will produce a more accurate and robust prediction model. However, participants may exhibit adaptability to a large number painful stimulation. For example, in our experiments, the correlation coefficient between pain ratings and trial numbers is significantly smaller than 0 (*R* = -0.17 ± 0.24, *P* = 1.95 × 10^-4^, one-sided *t*-test), which means that pain ratings decreased after a number of trials and the subjects may present adaptation to pain. Hence, it will be interesting to investigate why pain ratings are decreased with the trial numbers and whether pain-related BOLD responses also change with the trial numbers, which may lead to a better pain prediction model. In addition, it is necessary to deliver painful stimuli in a larger range of intensity so that the evoked pain ratings could also have a wider range, which will make class labels of the prediction model more complete and will lead to a more accurate and general prediction model.

## Ethics Statement

The protocol was approved by the ethics committee of Southwest University. All subjects gave written informed consent in accordance with the Declaration of Helsinki.

## Author Contributions

QL and LL analyzed the data. QL and JL collected the data. QL, WL, GH, and ZZ discussed the results and wrote the paper.

## Conflict of Interest Statement

The authors declare that the research was conducted in the absence of any commercial or financial relationships that could be construed as a potential conflict of interest. The handling Editor declared a past co-authorship with two of the authors GH and ZZ.

## Footnotes

1 http://www.fil.ion.ucl.ac.uk/spm/

2 https://www.mathworks.com/help/stats/plsregress.html
